# Oral Supplementation with L-Carnosine Attenuates Social Recognition Deficits in CD157KO Mice via Oxytocin Release

**DOI:** 10.3390/nu14040803

**Published:** 2022-02-14

**Authors:** Takahiro Tsuji, Kazumi Furuhara, Maria Gerasimenko, Anna Shabalova, Stanislav M Cherepanov, Kana Minami, Haruhiro Higashida, Chiharu Tsuji

**Affiliations:** 1 Research Center for Child Mental Development, Kanazawa University, Kanazawa 920-8640, Japan; furururukz.999@gmail.com (K.F.); mgera_08@mail.ru (M.G.); shabalova@gmail.com (A.S.); stas4476@mail.ru (S.M.C.); minami-k@staff.kanazawa-u.ac.jp (K.M.); haruhiro@med.kanazawa-u.ac.jp (H.H.); 2 Department of Ophthalmology, Faculty of Medical Sciences, University of Fukui, Fukui 910-1193, Japan; 3 Department of Health Development Nursing, Institute of Medical, Pharmaceutical and Health Sciences, Kanazawa University, Kanazawa 920-0934, Japan; 4 United Graduate School of Child Development, Osaka University, Kanazawa University, Hamamatsu University School of Medicine, Chiba University, and University of Fukui, Suita 565-0871, Japan

**Keywords:** L-carnosine, oxytocin, social behavior, autism spectrum disorder, social memory, oral supplements

## Abstract

The outcomes of supplementation with L-carnosine have been investigated in clinical trials in children with autism spectrum disorder (ASD). However, reports on the effects of L-carnosine in humans have been inconsistent, and the efficacy of L-carnosine supplementation for improving ASD symptoms has yet to be investigated in animal studies. Here, we examined the effects of oral supplementation with L-carnosine on social deficits in CD157KO mice, a murine model of ASD. Social deficits in CD157KO mice were assessed using a three-chamber social approach test. Oral supplementation with L-carnosine attenuated social behavioral deficits. The number of c-Fos-positive oxytocin neurons in the supraoptic nucleus and paraventricular nucleus was increased with L-carnosine supplementation in CD157KO mice after the three-chamber social approach test. We observed an increase in the number of c-Fos-positive neurons in the basolateral amygdala, a brain region involved in social behavior. Although the expression of oxytocin and oxytocin receptors in the hypothalamus was not altered by L-carnosine supplementation, the concentration of oxytocin in cerebrospinal fluid was increased in CD157KO mice by L-carnosine supplementation. These results suggest that L-carnosine supplementation restores social recognition impairments by augmenting the level of released oxytocin. Thus, we could imply the possibility of a safe nutritional intervention for at least some types of ASD in the human population.

## 1. Introduction

Autism spectrum disorder (ASD) is a neurodevelopmental disorder characterized by symptoms, such as impaired social communication and interaction as well as restricted, repetitive behavioral patterns. At present, there is a paucity of drugs that are able to ameliorate social deficits in individuals with ASD. Although the incidence of ASDs varies geographically, the global incidence is increasing every year [1].

Oxytocin (OT) plays diverse roles in mammalian social behaviors and has received growing interest as a potential therapy for various social and affective disorders. Evidence from animal studies has demonstrated the widespread role of OT in social recognition, attachment, parental behaviors, selective social bonding, anxiety, and stress coping [2,3]. In humans, intranasally administered OT affects social perception, improves discrimination of facial expressions [4,5], and increases gaze fixation on the eye region [6]. In addition, OT administration has been reported to enhance feelings of trust [7], empathy, and generosity [8]. Peripheral administration of OT has thus, been considered as a potential treatment for autism; however, clinical trials have reported inconsistent results [9]. This may be due to the lack of power in the study design and genetic heterogeneity of ASD, as well as the low permeability of the blood-brain barrier and lack of stable metabolism in plasma when OT is externally applied as a therapeutic agent. Accordingly, therapeutics that effectively increase endogenous OT would be beneficial in this regard.

L-carnosine is an imidazole dipeptide (B-alanyl-L-histidine) expressed abundantly in the muscles and brain of animals [10]. L-carnosine has diverse physiological functions, such as antioxidant properties, pH buffering, chelating of divalent cations, anti-glycation effects, and anti-convulsive properties [10]. In neurons, L-carnosine functions as an antioxidant and neuroprotectant [10]. L-carnosine is co-localized at glutamatergic synapses, preserves brain levels of the reduced form of glutathione, and attenuates inflammation and degeneration by reducing tumor necrosis factor-α (TNF-α) and nitric oxide synthesis [11,12]. L-carnosine, which is metabolized into histamine [13,14], inhibits glutamate release [15]. Further, L-carnosine acts as a gamma-aminobutyric acid (GABA) modulator, activates GABA receptors to exert neuroprotective effects on motor neuron excitotoxicity [16] and elicits anti-convulsive protection in vitro by increasing extracellular GABA levels [17]. These findings highlight the key role of L-carnosine in the modulation of excitatory/inhibitory balance in the brain, which may ameliorate brain dysfunction. Several reports have suggested that L-carnosine may be associated with OT function. For instance, supplementation with chicken breast extract, a commercially available carnosine-rich supplement, results in an accumulation of carnosine in the hippocampus and hypothalamus in rats [18]. Intraventricular administration of L-carnosine decreases food intake in chicks [18], which may be related to the potent central effects of OT on feeding behavior and anorectic effects [2,19].

The etiology of ASD is underscored by oxidative stress, decreased glutathione, excessive glutamate in plasma, and an imbalance between excitatory glutamate and inhibitory GABA signaling. Several studies have, therefore, investigated the therapeutic utility of L-carnosine supplementation in children with ASD. However, the results of small-scale clinical studies have been inconsistent, with some studies showing positive effects on social and behavioral responses [20] and others reporting no positive effects [21], highlighting the need for further investigations on a larger scale [22].

To date, there has been a lack of basic studies examining the effects of L-carnosine using animal models of ASD, and evidence supporting the effectiveness of L-carnosine supplementation for treating ASD symptoms, especially deficits in social behaviors, is scarce. Animal models of psychiatric disorders are essential for elucidating neural mechanisms and predicting the efficacy of therapeutic agents in preclinical settings. Accordingly, it is crucial to evaluate the impact of L-carnosine on ASD symptoms using animal models of ASD and to clarify the site(s) of action of L-carnosine.

CD157 was first identified as bone marrow stromal antigen-1 in cell lines from patients with rheumatoid arthritis [23]. CD157 plays a key role in the immune system [24,25,26] and has been reported as a risk factor for neurodegeneration, particularly Parkinson’s disease [27,28,29,30]. Although little is known about the physiological roles of CD157 in the brain, or the context of brain degeneration in Parkinson’s disease, an association between the *CD157/BST1* gene and ASD has been reported [31,32]. Homozygous CD157 knockout (*CD15*7KO) mice display social behavioral impairments and anxiety-related and depression-like behaviors that are reversed by treatment with anti-depressant drugs and OT [33,34,35]. These findings suggest that CD157KO mice may be useful as a mouse model of ASD with regard to modeling behaviors associated with ASD symptoms.

The aim of this study was to examine the effects of carnosine supplementation on social recognition, which is deficient in ASD, using CD157KO mice as a murine model of ASD. The effects of long-term supplementation with L-carnosine on social recognition were assessed using the three-chamber social approach test. Further, we explored the effects of L-carnosine supplementation on OT signaling, which plays a critical role in diverse social behaviors.

## 2. Materials and Methods

### 2.1. Animals

C57BL6/N wild-type (WT) mice were purchased from Japan SLC Inc. (Hamamatsu, Japan) via Sankyo Laboratory Service Corporation (Toyama, Japan). CD157KO mice were generated as previously described [36]. CD157KO mice were generated for experiments by breeding homozygous mutant mice. WT and CD157KO mice were kept at the Institute for Experimental Animals, Advanced Science Research Center, Kanazawa University, under controlled conditions (22 °C; 12-h light/dark cycle, lights on at 8:45 a.m.) and in standard mouse cages (300 × 160 × 110 mm) with sawdust bedding. Food and water were supplied *ad libitum*. Mice were weaned at 21–28 days of age and kept in same-sex groups of 3–5 animals until 11 weeks old. Then, male mice were separated into individual cages for 14 days before carrying out the behavioral experiments. L-carnosine-treated WT or CD157KO mice were maintained on a constant dosage of L-carnosine diluted in drinking water (FUJIFILM Wako Chemicals, Osaka, Japan, 0.09 g/100 mL) from weaning (3 week-old) until behavioral testing (13 week-old). The number of mice used in the behavioral tests is 16–19 per group, 7–13 per group for immunostaining, and 19–28 per group for CSF measurement. The number of animals used in each data is written in the graph of figures. Chronic treatment with L-carnosine did not significantly alter water consumption, food consumption, and the bodyweight of mice in the wild-type (WT) and CD157KO groups treated with (WT-C, CD157KO-C) or without (WT, CD157KO) L-carnosine-containing water. This study was approved by the Committee on Animal Experimentation of Kanazawa University (AP-143261) and carried out according to the Fundamental Guidelines for Proper Conduct of Animal Experiments and Related Activities in Academic Research Institutions under the jurisdiction of the Ministry of Education, Culture, Sports, Science and Technology of Japan.

### 2.2. Three-Chamber Social Approach Test

The behavior tests were carried out using a three-chamber box as previously reported [33,37]. Briefly, the apparatus consisted of a rectangular, three-chambered box covered with transparent polycarbonate. There were doorways in the dividing walls, allowing mice to freely enter and exit each room. An empty wire mesh cage (70 × 90 × 70 mm) was placed on each side. At the end of each test, the apparatus was wiped with 1% sodium hypochlorite followed by 70% ethanol to remove odor. The following procedure was used for the social behavior test: (A) Habituation. The test mouse was set in the middle chamber and allowed to explore all parts of the arena for 20 min a day before the test and 10 min pre-test. (B) Sociability test. After 10 min of habituation, an unfamiliar mouse (Stranger 1; a naïve C57BL6/N male) was set in the wire cage of the left chamber and an object was placed in the right chamber. The test mouse was set in the center compartment to explore for 5 min. (C) Social preference test. The test mouse was temporarily removed from the chamber. An unfamiliar mouse was set in the wire cage of the right chamber (Stranger 2). The test mouse was then set again in the middle chamber and allowed to explore for 5 min. The amount of time spent near the wire cage zone (within 2 cm from the wire cage) was measured using a digital video system and ANY-maze software (Stoelting Co, Wood Dale, IL, USA). The sociability index was calculated as follows: delta (the interaction time spent with an object was subtracted from the interaction time spent with Stranger 1) was divided by the total time spent with an object and Stranger 1. The social preference index was calculated as follows: delta (the interaction time spent with Stranger 2 was subtracted from the interaction time spent with Stranger 1) was divided by the total time spent with both stranger mice. 

### 2.3. Real-Time Reverse Transcriptase Quantitative PCR

Total RNA was extracted from the tissues using TRIzol Reagent (Life Technologies, Carlsbad, CA, USA). cDNA was synthesized from 1.0 μg of total RNA using the ReverTra Ace α cDNA Synthesis Kit (Toyobo, Osaka, Japan) according to the manufacturer’s protocol. Then, the real-time reverse transcriptase quantitative PCR (RT-qPCR) analysis was performed using a cDNA template with ViiA™7 Real-Time PCR System (Applied Biosystems, Foster City, CA, USA). Each sample was quantified in duplicate in a 20 μL amplification reaction mixture containing 10 μL Taqman Fast Universal PCR Master Mix (4352042, Applied Biosystems), 1 μL TaqMan Gene Expression Assays, which includes a set of unlabeled PCR primers and one TaqMan probe (OT, Mm01329577_g1 or OT receptor, Mm01182684_m1, Applied Biosystems), 3 μL cDNA template, and 6 μL nuclease-free water. The values obtained for the target genes were normalized against glyceraldehyde 3-phosphate dehydrogenase mRNA expression (TaqMan Gene Expression Assays, Mm99999915_g1, Applied Biosystems) and calculated using the 2^−ΔΔCt^ method. The relative values were calculated based on the values of the WT group.

### 2.4. Tissue Preparation

Mice were anesthetized with sodium pentobarbital (200 mg/kg body weight, i.p.) 70 min after the social preference tests. Then, mice were transcardially perfused with a heparinized (20 U/mL) 0.9% saline solution followed by 4% paraformaldehyde in 0.1 mol/L phosphate buffer (PB). The removed brains were immersed in a solution of 4% paraformaldehyde containing 15% sucrose in 0.1 mol/L PB at 4 °C. Subsequently, they were put into a solution of 30% sucrose in 0.1 mol/L PB and incubated for at least 72 h before processing. The brains were cut at 40 μm (coronal) using a freezing microtome and stored in a cryoprotectant solution (30% ethylene glycol + 20% glycerol in 0.05 mol/L sodium phosphate buffer, pH 7.3) at 4 °C until further use.

### 2.5. Immunocytochemistry

The coronal sections were rinsed in PB for 5 min, three times to remove excess fixative/cryoprotectant. Sections were permeabilized in washing buffer (0.3% Triton X-100 in PB) for 20 min followed by pre-incubation in blocking solution (3% goat serum and 0.3% Triton X-100 in PB) for 1 h at room temperature (20–25 °C). Then, sections were incubated with a rabbit anti-c-Fos polyclonal antibody (1:1000, 226003, Synaptic Systems, Göttingen, Germany) and mouse anti-OT monoclonal antibody (1:200, PS38, CRL-1950, American Type Culture Collection, Manassas, VA, USA) diluted in the blocking solution. After incubating 36 h at 4 °C, sections were washed five times with washing buffer for 5 min, then incubated for 60 min at room temperature with goat anti-mouse IgG antibody conjugated with Alexa Fluor 488 (1:200, A-11001, Thermofisher Scientific, Waltham, MA, USA), goat anti-rabbit IgG antibody conjugated with Alexa Fluor 594 (1:200, A-11012, Thermofisher Scientific) and 4′, 6-diamidino-2-phenylindole (1:2000, DAPI, D523, Dojindo, Kumamoto, Japan, 1:2000) diluted in PB. The sections were mounted on glass slides with PermaFluor Aqueous Mounting Medium (TA-030-FM, Thermo Scientific, Kalamazoo, MI, USA) after washing three times in PB for 5 min.

### 2.6. Image Quantification

Images were acquired using an Olympus IX71 inverted microscope (Olympus Corporation, Tokyo, Japan) equipped with a cooled CCD camera (Cool SNAP HQ2; Roper Scientific, Tucson, AZ, USA) and Olympus UPlan Apo 0.4 NA x10 objective (Olympus). Brain structures were anatomically identified according to the atlas of Franklin and Paxinos (1997). Sections used for quantification were as follows: supraoptic nucleus (SON) and paraventricular nucleus (PVN) of the hypothalamus (bregma between −0.82 mm and −0.94 mm anterior-posterior (AP)), basolateral amygdala (BLA) (bregma between −1.22 mm and −1.34 mm, AP), posteroventral medial amygdala (MePV) (bregma between −1.58 mm and −1.70 mm, AP). Image analysis was performed using ImageJ (NIH, Bethesda, MD, USA). For PVN, the number of c-Fos immune-positive nuclei (c-Fos) was counted in the region enclosed by the dotted square (shown in Figure 2A) at a distance of approximately 100 μm apart from the third ventricle. For SON, the number of c-Fos was counted within the area enclosed by the dotted line (shown in Figure 2A) where the OT neurons reside. For BLA and MePV, the c-Fos was counted in the region of interest (ROI) which was placed within the dotted line shown in Figure 3B with the almost same size for every section. The number of c-Fos was normalized by the area used for counting. The percentage of c-Fos-positive OT neurons was defined as the number of c-Fos-positive OT neurons over the total number of OT neurons. The number of OT neurons was counted within the same area used for counting c-Fos. To quantify the expression of OT, the intensity of each OT neuron’s cell body was measured. The cell body of OT neurons residing in the half hemisphere of PVN or SON were measured and averaged. The counts from two to three brain sections per mouse were averaged and represented as each value in the graph. The investigators were blinded to the treatment groups when performing cell counts.

### 2.7. Sampling of Cerebrospinal Fluid (CSF)

CSF samples were collected without detectable plasma contamination according to the protocol with some modifications [38]. Briefly, mice were anesthetized by sodium pentobarbital anesthesia (100 mg/kg, i.p.) and the occipital skull was exposed. The dura mater of the cisterna magna was visualized as a glistening and clear reverse triangle through which the medulla oblongata, a major blood vessel (arteria dorsalis spinalis), and CSF space were visible. A capillary tube was inserted into the cisterna magna through the dura mater, and the blood-free CSF was sampled with a 32G needle. The samples were immediately frozen on dry ice and stored at −80 °C until quantification.

### 2.8. Enzyme Immunoassay for OT

OT immunoreactivity levels in CSF were quantified using an oxytocin EIA kit (ADI-901-153A-0001, Enzo Life Sciences, NY, USA; formerly Assays Designs, MI, USA), according to the manufacturer’s protocol. Thawed CSF samples (5 μL) were diluted at a 1:20 ratio in assay buffer. Due to the low protein concentration in the CSF, samples were quantified without protein extraction. The OT assay had two linear ranges, including a lower concentration range from 15 pg/mL to 30 pg/mL and a higher concentration range between 50 and 1000 pg/mL. The inter-assay and intra-assay coefficients of variation were less than 15%.

### 2.9. Statistical Analysis

Statistical analysis was performed using Prism 8 software (GraphPad Software Inc., San Diego, CA, USA). The data are presented as mean ± SEM. Two-way analysis of variance (ANOVA) was used to assess the effects of genotype (WT or CD157KO) × treatment (water or L-carnosine), followed by posthoc analysis with Bonferroni’s multiple comparison test. A two-tailed paired Student’s t-test was used to compare the interaction time in the three-chamber social approach test.

## 3. Results

### 3.1. Effects of L-Carnosine Supplementation on Social Behavior

**Figure 1:**
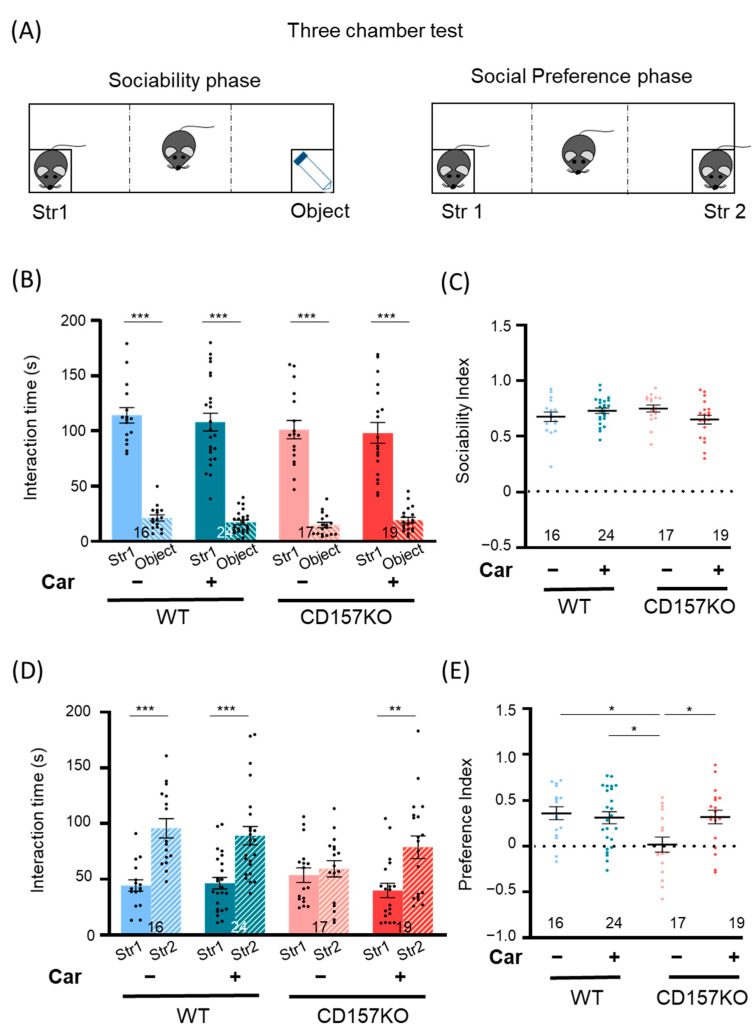
Social behavioral deficits in CD157KO mice were improved by chronic L-carnosine intake. (**A**) Experimental scheme of the three-chamber social recognition test. The social preference test was performed after the sociability test. (**B**) The sociability test. The time spent around the wire cage with a social target (Stranger 1) or with an object was measured. (**C**) The sociability index. (**D**) The social preference test. The time spent around the wire cage with a familiar social target (Stranger 1) or with an unfamiliar social target (Stranger 2) was measured. (**E**) The social preference index. The numbers of mice used in each experiment are indicated in the graphs. Data are presented as the mean ± SEM. * *p* < 0.05, ** *p* < 0.01, *** *p* < 0.001. WT: wild-type, Str: stranger, Car, L-carnosine.

CD157KO male mice demonstrate deficits in social recognition [33,37]. We, therefore, examined the effect of long-term supplementation with L-carnosine on social recognition in the three-chamber social preference test (Figure 1A). L-carnosine was supplemented by addition to drinking water from weaning to 13 weeks of age. As previously reported, wild-type (WT) and CD157KO (CD157KO) groups spent a longer time with mice (Stranger 1) than with the object (Figure 1B) [33]. L-carnosine treatment did not affect preference for the social target in both L-carnosine-treated wild-type (WT-C) and CD157KO (CD157KO-C) groups (Figure 1C). In the social preference test, WT and WT-C mice spent twice as long with the novel mouse (Stranger 2) than with the familiar mouse (Stranger 1) (Figure 1D, WT, and WT-C, both *p* < 0.001, paired *t*-test). In contrast, CD157KO mice spent almost equal time with the familiar mouse (Stranger 1) and the novel mouse (Stranger 2) (Figure 1D, CD157KO, *p* = 0.526, paired *t*-test). In CD157KO mice, L-carnosine treatment reversed the lack of preference toward the novel mouse to a level similar to that of WT mice (Figure 1D, CD157KO-C, *p* = 0.003, paired *t*-test). To evaluate the strength of social preference, we assessed the preference index (Figure 1E). Two-way ANOVA revealed a significant effect of genotype (F (1, 72) = 5.16, *p* = 0.026) and a significant interaction between genotype and treatment (F (1, 72) = 5.63, *p* = 0.020). Posthoc Bonferroni’s multiple comparison tests revealed a significant difference between WT and CD157KO mice (*p* = 0.017), WT-C and CD157KO mice (*p* = 0.028), and CD157KO and CD157KO-C mice (*p* = 0.035), but not between WT and CD157KO-C mice (*p* > 0.999). These data indicated that social deficits in CD157KO mice were improved by L-carnosine supplementation.

### 3.2. c-Fos Activation in the SON and PVN

**Figure 2:**
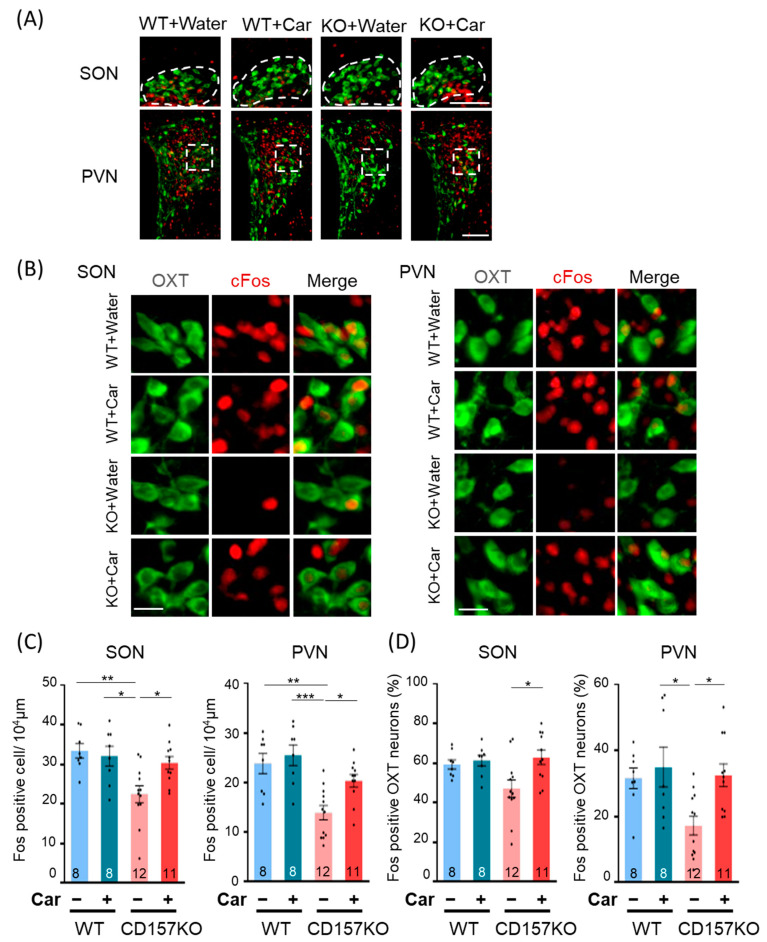
Induction of c-Fos in the supraoptic nucleus (SON) and paraventricular nucleus (PVN) of the hypothalamus after social recognition tests. (**A**) Representative merged immunostaining images of oxytocin (OT) (green) and c-Fos (red) in the SON and PVN in low magnification. The dotted areas are used for counting. Scale bars, 100 μm. (**B**) Representative immunostaining images of OT (green, left), c-Fos (red, middle) and merge (right) in high magnification. Scale bars, 20 μm. (**C**) The number of c-Fos-positive cells in the SON and PVN. (**D**) The percentage of c-Fos-expressing OT neurons. The percentages represent the number of c-Fos-positive OT neurons over the total number of OT neurons. The number of mice used for quantification in each group is indicated in the graph. Data are presented as the mean ± SEM. * *p* < 0.05, ** *p* < 0.01, *** *p* < 0.001. WT: wild-type, Car: L-carnosine.

Previous reports have demonstrated that social behavioral deficits in CD157KO mice are ameliorated by intraperitoneal administration of OT [33], and OT is expressed predominantly in the SON and PVN of the hypothalamus [2]. We, therefore, investigated the effects of L-carnosine supplementation on neuronal activity in the PVN and SON. Brain tissues were collected after the social preference test and subjected to immunohistochemistry to detect c-Fos, a neuronal activation marker. In the SON, the number of c-Fos-positive neurons was lower in CD157KO mice (22.48 ± 2.13/10^4^ μm^2^) than in WT mice (33.42 ± 1.80/10^4^ μm^2^), but the number of c-Fos-positive neurons in mice that received L-carnosine supplementation was similar to that in WT mice (CD157KO-C, 30.38 ± 1.59/10^4^ μm^2^, Figure 2A–C). Two-way ANOVA revealed a significant interaction of genotype and treatment interaction (F (1, 35) = 5.061, *p* = 0.031) and a significant main effect of genotype (F (1, 35) = 9.476, *p* = 0.004). Posthoc analysis with Bonferroni’s multiple comparison test revealed significant differences between the WT and CD157KO groups (*p* = 0.003), CD157KO and CD157KO-C groups (*p* = 0.030), and WT-C and CD157KO groups (*p* = 0.012). The number of c-Fos-positive neurons in the PVN was lower in CD157KO mice (13.93 ± 1.44/10^4^ μm^2^) than in WT mice (23.84 ± 2.03/10^4^ μm^2^), similar to the results observed for the SON (Figure 2A–C). L-carnosine supplementation increased the number of c-Fos-positive neurons to WT levels (CD157KO-C, 20.38 ± 1.29/10^4^ μm^2^). Two-way ANOVA revealed a significant effect of genotype (F (1, 35) = 15.90, *p* < 0.001) and treatment (F (1, 35) = 4.184, *p* = 0.048). Posthoc analysis with Bonferroni’s multiple comparison test revealed significant differences between the WT and CD157KO groups (*p* = 0.002), CD157KO and CD157KO-C groups (*p* = 0.047), and WT-C and CD157KO groups (*p* < 0.001). These results indicated that neuronal activation in the SON and PVN was lower in CD157KO mice than in WT mice, but L-carnosine supplementation resulted in an increase in neuronal activation in CD157KO mice to the levels observed in WT mice. Nevertheless, neuronal activation in WT mice was not affected by L-carnosine supplementation.

Next, to examine potential differences in the activity of OT neurons following the social preference test, we assessed the percentage of c-Fos-positive OT neurons in the SON and PVN. In both regions, the percentage of c-Fos-positive OT neurons was higher in the CD157KO-C group than in the CD157KO group (Figure 2A,B,D). Accordingly, for the SON, two-way ANOVA revealed a significant effect of treatment (F (1, 35) = 5.342, *p* = 0.027), and posthoc analysis with Bonferroni’s multiple comparison test revealed a significant difference between the CD157KO and CD157KO-C groups (*p* = 0.017). For the PVN, two-way ANOVA revealed a significant effect of treatment (F (1, 35) = 5.363, *p* = 0.027) and genotype (F (1, 35) = 4.332, *p* = 0.045). Posthoc analysis with Bonferroni’s multiple comparison test revealed a significant difference between the CD157KO and CD157KO-C groups (*p* = 0.028) and a trend for less OT neuronal activation in the CD157KO group than in the WT group (*p* = 0.081). These findings collectively suggested that L-carnosine treatment resulted in significant activation of OT neurons in the SON and PVN in CD157KO mice.

### 3.3. Effects of L-Carnosine Supplementation on c-Fos Expression in the BLA and MePV in CD157KO Mice

**Figure 3:**
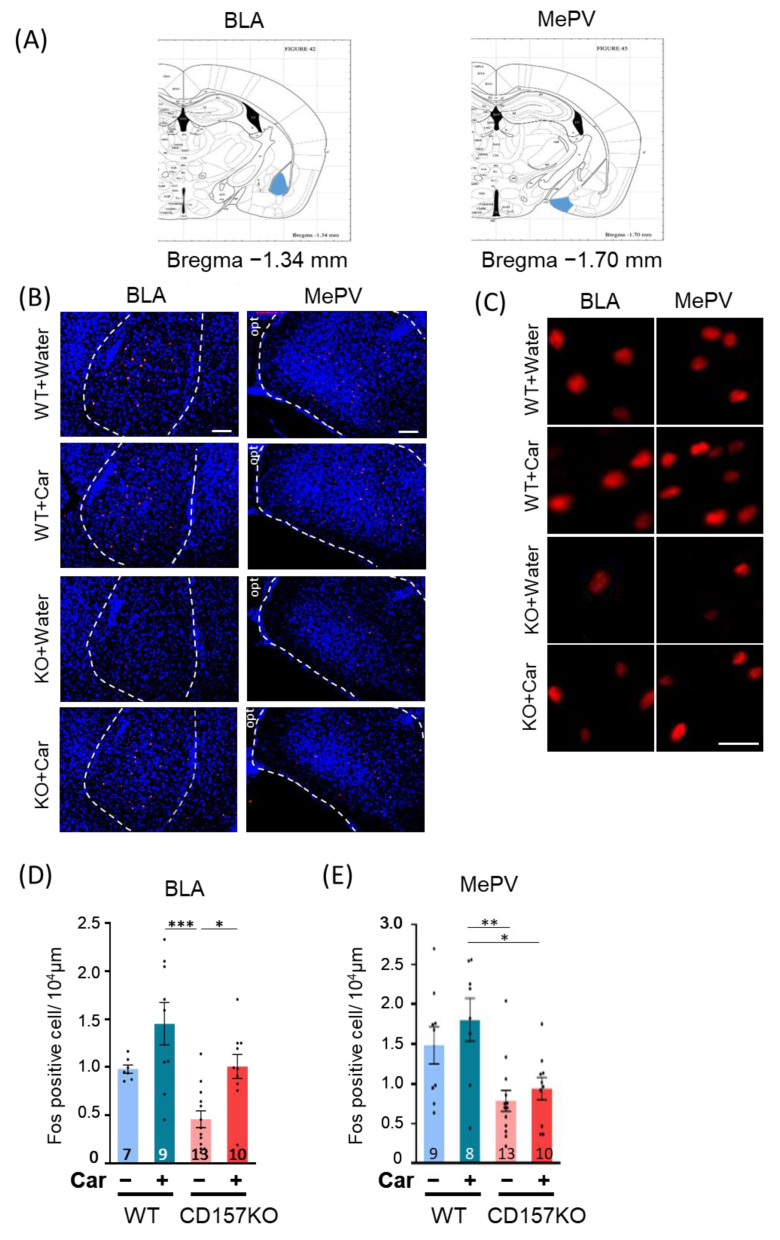
Induction of c-Fos in the basolateral amygdala (BLA) and posteroventral medial amygdala (MePV) after social recognition tests. (**A**) The brain regions used for quantification of c-Fos in the BLA and MePV are shown in blue. (**B**,**C**) Representative immunostaining images of c-Fos in the BLA and MePV in low magnification ((**B**), c-Fos in red and 4′, 6-diamidino-2-phenylindole (DAPI) in blue, scale bars, 100 μm) and high magnification (**C**, c-Fos in red, scale bar, 20 μm). The dotted areas are used for counting. (**D**,**E**) The number of c-Fos-positive cells in the BLA (**D**) and MePV (**E**). The number of mice used for quantification in each group is indicated in the graph. Data are presented as the mean ± SEM. * *p* < 0.05, ** *p* < 0.01, *** *p* < 0.001. WT: wild-type, Car: L-carnosine.

We next examined whether increased activation of OT neurons modulated neuronal activity in brain regions receiving OT input. We examined c-Fos induction in functionally distinct subdivisions of the amygdala that express OT receptors. In the BLA, c-Fos is induced by conspecific social cues [39], whereas in the MePV, c-Fos is induced by reproduction-related behaviors [40,41]. The number of c-Fos-positive cells was examined after the social preference test (Figure 3B,C). For the BLA, two-way ANOVA revealed a significant effect of genotype (F (1, 35) = 12.26, *p* = 0.0013) and treatment (F (1, 35) = 13.71, *p* < 0.001) (Figure 3D). Posthoc analysis with Bonferroni’s multiple comparison test revealed that the number of c-Fos-positive cells was higher in the CD157KO-C group than in the CD157KO group (*p* = 0.023). The number of c-Fos-positive cells in the CD157KO group tended to be lower than that in the WT group, but this did not reach statistical significance (*p* = 0.074). For the MePV, two-way ANOVA revealed a significant effect of genotype (F (1, 36) = 17.03, *p* < 0.001), but no significant difference was detected between CD157KO and CD157KO-C groups in posthoc analysis with Bonferroni’s multiple comparison test (Figure 3E). WT and CD157KO groups exhibited a trend to decrease (*p* = 0.059). Furthermore, L-carnosine treatment did not reach significance for the induction of c-Fos positive cells in WT in both brain regions (Figure 3D,E). These results suggested that L-carnosine supplementation resulted in an increase in neuronal activity in the BLA, but not in the MePV of CD157KO mice to the same extent as that in WT mice after the social preference test. These data indicated that L-carnosine supplementation resulted in the activation of brain regions involved in downstream OT signaling in the context of social recognition.

### 3.4. L-Carnosine Treatment Increased Oxytocin Concentration in CSF

**Figure 4:**
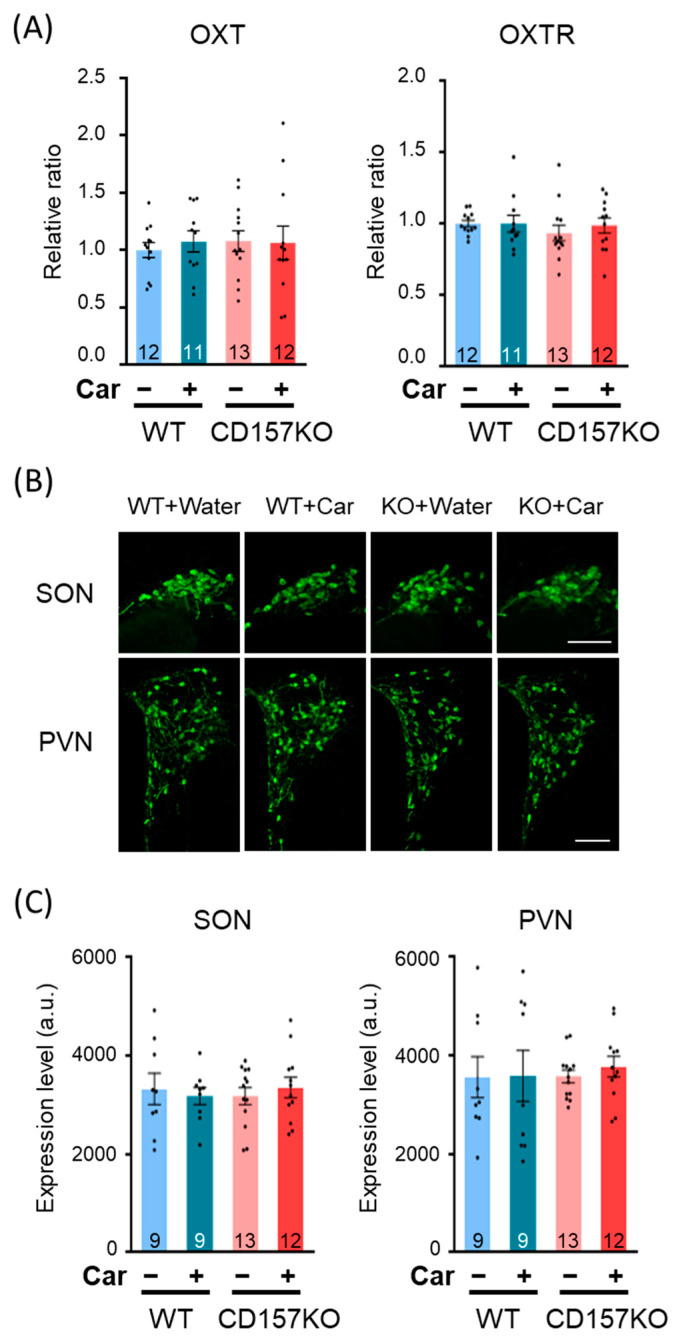
The expression levels of OT and OT receptors in the SON and PVN. (**A**) Relative mRNA expression of OT and OT receptors in the hypothalamus. (**B**) Representative images of OT immunostaining in the SON and PVN. (**C**) OT expression in the SON and PVN. The intensity of OT immunofluorescence was measured. Data are presented as the mean ± SEM. The number of mice used for quantification in each group is presented in the graph. WT: wild-type, Car: L-carnosine. Scale bars, 100 μm.

Given that L-carnosine supplementation resulted in an increase in OT neuronal activation, we investigated the effects of L-carnosine supplementation on the expression of OT and OT receptors. No significant differences were detected in mRNA expression levels of OT and OT receptors in the hypothalamus among groups (Figure 4A). To examine OT protein expression levels, we measured the fluorescence intensity of OT neurons in the SON and PVN using immunofluorescence histochemistry (Figure 4B,C). No significant differences were detected in the intensity of OT immunofluorescence among groups.

**Figure 5:**
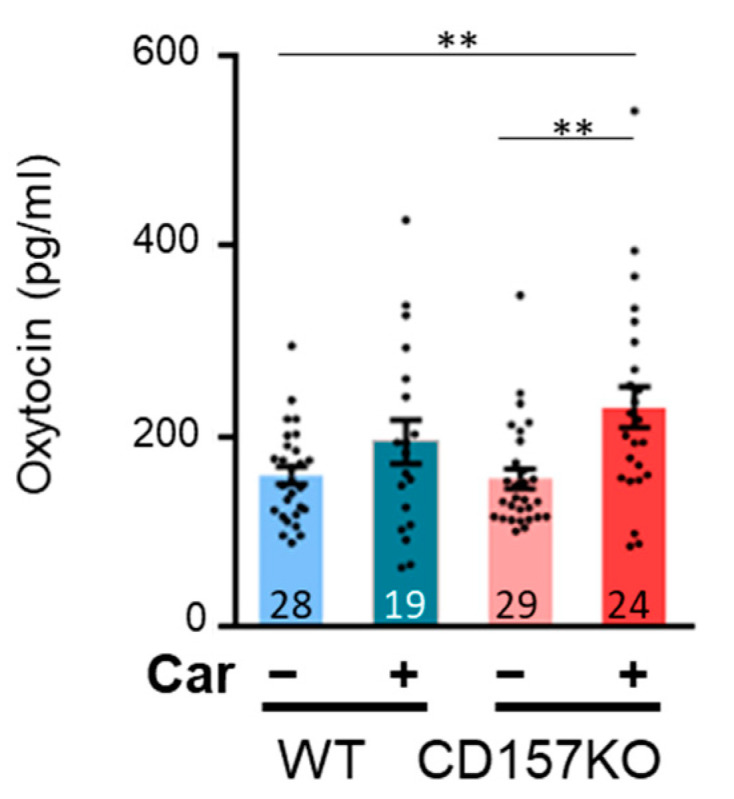
The concentration of OT in cerebrospinal fluid (CSF). Data are presented as the mean ± SEM. ** *p* < 0.01 The number of mice used for quantification in each group is indicated in the graph. WT: wild-type, Car: L-carnosine.

We subsequently investigated the effects of L-carnosine supplementation on OT levels in CD157KO mice by measuring the basal concentration of OT in CSF. The OT concentration in L-carnosine-treated CD157KO mice was higher than that in CD157KO or WT mice. Two-way ANOVA revealed a significant effect of treatment (F (1, 96) = 12.03, *p* < 0.001, Figure 5). Posthoc analysis with Bonferroni’s multiple comparison test revealed a significant difference between the CD157KO-C and WT and/or CD157 groups (CD157KO-C vs. WT, *p* = 0.008; CD157KO-C vs. CD157KO, *p* = 0.005). These results suggested that OT release was increased after L-carnosine supplementation in CD157KO mice.

## 4. Discussion

In this study, we observed that L-carnosine intake improved social recognition memory in CD157KO mice, a mouse model of ASD. L-carnosine supplementation did not significantly alter the expression levels of OT or OT receptors in the hypothalamus. However, the concentration of OT in CSF was significantly higher in L-carnosine-supplemented CD157KO mice than in non-supplemented mice. In accordance with previous findings indicating that OT administration rescued deficits in social behavior in CD157KO mice, increased concentration of released OT may have ameliorated the social deficits observed in CD157KO mice.

CD157 and CD38 belong to a family with ADP-ribosyl cyclase activity which converts NAD(+) into cyclic ADP-ribose (cADPR), a potent intracellular Ca^2+^ mobilizer [42,43]. cADPR production by CD157 is not as high as that by CD38, and the expression of CD157 in the brain in adulthood is lower than that of CD38, although weak CD157 immunoreactivity can be detected in the amygdala. CD157 is more strongly expressed during the embryonic stage compared to that during the early postnatal period, and it gradually decreases by postnatal day 7 [35]. In CD157KO mice, the amygdala, a key node in social brain circuits, is altered and the impairment in neuronal activation is shown [33]. Therefore, the loss of CD157 during developmental stages may have resulted in differential development of the neural circuits underlying social behavior in CD157KO mice compared to that in WT mice. However, further analysis is necessary to elucidate the mechanisms underlying the recovery of social behavioral deficits induced by increased levels of endogenous OT or by external application of OT.

The physiological roles of L-carnosine in the brain associated with OT signaling remain unclear, although preliminary findings have been reported. For example, supplementation with chicken breast extract, a commercially available carnosine-rich supplement, results in an accumulation of carnosine in the hippocampus and hypothalamus in rats [18]. Intraventricular administration of L-carnosine in chickens decreases food intake [44], which may be related to the potent central effects of OT on feeding behavior and anorectic effects [2,19]. Moreover, L-carnosine plays a well-established role in skeletal muscle and cardiac contraction [10]. L-carnosine application induces rapid Ca^2+^ release from the sarcoplasmic reticulum and is suggested to modulate the activity of ryanodine receptors [45]. The function of L-carnosine in the brain may be similar to that in peripheral tissues, whereby it promotes an increase in cytosolic Ca^2+^ in OT neurons. A key molecular signaling event involved in OT release is the mobilization of Ca^2+^ from intracellular stores, Ca^2+^-induced Ca^2+^-release, which involves cADPR [42]. cADPR, catalyzed by the CD38 enzyme, facilitates an increase in free cytosolic Ca^2+^ concentration via ryanodine receptor-sensitive Ca^2+^ pools [43]. CD38 knockout mice exhibit low OT levels in plasma and CSF and exhibit impairments in social behaviors [46]. In this regard, L-carnosine may be involved in OT release via the ryanodine receptor.

OT has been reported to act on connections between the prefrontal cortex and amygdala. This pathway is known to regulate sociality in rodents and primates, including humans [47]. It may form the basis for therapeutic targets of ASD and other social disorders associated with OT signaling. In mice, the neural pathway from the medial prefrontal cortex to BLA is involved in social recognition [47], and c-Fos is induced in the BLA after social recognition experiments [39]. Furthermore, the pathway from the BLA to the infralimbic cortex or prelimbic cortex has been implicated in social approach-avoidance behavior using the Targeted Recombination in Active Populations method [48]. In contrast, the MePV and bed nucleus of the stria terminalis are involved in reproductive behaviors [49]. In the present study, we measured the number of c-Fos-positive cells in the MePV and BLA, two functionally distinct amygdaloid regions that express OT receptors. Increased OT levels were observed in L-carnosine-treated CD157KO mice alongside induction of c-Fos in the BLA, but not in the MePV. Our results may reflect differences in behavior-related neuronal activation.

Using a mouse model of ASD, we demonstrate that L-carnosine supplementation may ameliorate social deficits, a characteristic symptom of ASD. Cognitive function in individuals with brain disorders, including cerebrovascular, neurodegenerative, and neurodevelopmental disorders, may be improved by L-carnosine supplementation [29]. Studies have reported protective effects of L-carnosine in neurological and psychiatric disorders, Parkinson’s disease, Alzheimer’s disease, schizophrenia, obsessive-compulsive disorder, and attention-deficit/hyperactivity disorder [50,51,52,53]. However, clinical trials of L-carnosine supplementation in children with ASD have demonstrated inconsistent results [22]. Not all studies conducted to date have reported evident improvements in social dysfunction in children with ASD following L-carnosine supplementation. The discrepancies in the findings on L-carnosine supplementation in mice and humans may be due to the existence of carnosinase-1, a dipeptidase that degrades L-carnosine in human plasma, which is not expressed in rodents [54]. The stability of serum L-carnosine in rats is much higher compared to humans [55]. Therefore, simply providing L-carnosine supplementation may be insufficient to induce tangible effects on cognitive processes in humans. Accordingly, other approaches for administering L-carnosine in humans should be considered.

Recent studies have demonstrated that episodic memory in healthy elderly individuals and elderly individuals with mild cognitive impairments was improved by oral supplementation with the L-carnosine mixture comprising L-carnosine and anserine, a methylated form of carnosine [50,56]. The anserine is less readily degraded than L-carnosine in humans [57] and the mixture may thus result in positive effects on psychiatric symptoms. L-carnosine and anserine are both available on the market and have not been associated with any adverse events. Therefore, supplementation with L-carnosine and anserine may afford a novel therapeutic option for targeting oxytocin secretion in patients with ASD. Further studies are warranted to examine if anserine shares physiological properties and exerts similar effects on psychiatric symptoms compared to L-carnosine in clinical and preclinical settings.

In the future, we will examine the functional role of L-carnosine on OT release and the potential benefit of L-carnosine administration in other ASD models. In addition, we will explore the possibility that L-carnosine and/or anserine may be a safe nutritional intervention for at least some types of ASD in the human population.

## 5. Conclusions

In conclusion, we demonstrated that L-carnosine supplementation ameliorated deficits in social recognition in CD157KO mice. Social deficits in CD157KO mice were improved by oral supplementation with L-carnosine via an increase in OT concentration in CSF, which highlights the possibility of treating social deficits in individuals with ASDs. Our study is the first to provide preclinical evidence supporting the beneficial effects of L-carnosine supplementation on social deficits that have been reported in clinical studies.

## Data Availability

The data that support the findings of this study are available from the corresponding author upon reasonable request.
